# Encapsulation of Blackberry Phenolics and Volatiles Using Apple Fibers and Disaccharides

**DOI:** 10.3390/polym14112179

**Published:** 2022-05-27

**Authors:** Mirela Kopjar, Ivana Buljeta, Mario Nosić, Ivana Ivić, Josip Šimunović, Anita Pichler

**Affiliations:** 1 Faculty of Food Technology Osijek, Josip Juraj Strossmayer University of Osijek, F. Kuhača 18, 31000 Osijek, Croatia; ivana.buljeta@ptfos.hr (I.B.); m.nosic@yahoo.com (M.N.); ivana.ivic@ptfos.hr (I.I.); anita.pichler@ptfos.hr (A.P.); 2 Department of Food, Bioprocessing and Nutrition Sciences, North Carolina State University, Raleigh, NC 27695, USA; simun@ncsu.edu

**Keywords:** apple fiber, trehalose, sucrose, blackberry phenolics, blackberry volatiles

## Abstract

The objective of this study was to determine the effect of disaccharides on the encapsulation of the phenolics and volatiles of blackberry juice with the use of apple fiber. For this purpose, apple fiber/blackberry microparticles were prepared as the control, as well as microparticles additionally containing disaccharides, i.e., sucrose or trehalose. Fiber:disaccharide ratios were 1:0.5, 1:1, and 1:2. Formulated microparticles were characterized for total phenolics, proanthocyanidins, individual phenolics, antioxidant activity, flavor profiles, and color parameters. Both applied disaccharides affected the encapsulation of phenolics and volatiles by the apple fibers. Control microparticles had a higher content of phenolics than microparticles with disaccharides. Comparing disaccharides, the microparticles with trehalose had a higher content of phenolics than the ones containing sucrose. The amount of proanthocyanidins in the control microparticles was 47.81 mg PB2/100 g; in trehalose, the microparticles ranged from 39.88 to 42.99 mg PB2/100 g, and in sucrose, the microparticles ranged from 12.98 to 26.42 mg PB2/100 g, depending on the fiber:disaccharide ratio. Cyanidin-3-glucoside was the dominant anthocyanin. Its amount in the control microparticles was 151.97 mg/100 g, while in the trehalose microparticles, this ranged from 111.97 to 142.56 mg /100 g and in sucrose microparticles, from 100.28 to 138.74 mg /100 g. On the other hand, microparticles with disaccharides had a higher content of volatiles than the control microparticles. Trehalose microparticles had a higher content of volatiles than sucrose ones. These results show that the formulation of microparticles, i.e., the selection of carriers, had an important role in the final quality of the encapsulates.

## 1. Introduction

Color and flavor are very important quality parameters of foods, and they are very often the driving forces for consumers’ acceptance of particular food products. Nowadays, the utilization of different food additives is on the rise in order to enhance products with the use of color and/or flavor compounds. Furthermore, these compounds can have important health benefits, i.e., by making food additives functional. Fruits are rich in different bioactive and flavor compounds that make them very popular among consumers, but on the other hand, they are quite fragile. The high post-harvest respiration rate of fruits results in their nutritional and microbiological deterioration and limited shelf life. All of this leads to a loss of quality and health benefits. Traditionally, to avoid these negative aspects of post-harvest respiration, fruits are converted into different products (such as frozen, dried, canned products, jams, jellies, and juices) which can be available to consumers all year long. Nowadays, one of the emerging trends in the food industry is the formulation of delivery systems that can effectively preserve both phenolic and volatile compounds and efficiently incorporate them into food products. For that purpose, the selection of carriers/polymers for these compounds is of major importance. Nowadays, sustainability is quite an important subject in the food industry. Understanding the impact of synthetic materials on the environment directed the food industry toward natural polymers that can be beneficial as efficient eco-friendly materials [1]. The production of different products from apples leads to the formation of a considerable amount of its by-product, i.e., apple pomace, which can be used as a sustainable source for the production of valuable polymers such as apple fibers [2]. Dietary fibers have many health benefits, which makes them an excellent choice as delivery systems of selected active compounds. Some health benefits of fibers that have been highlighted in studies over the years are the lowering of blood cholesterol and sugar levels as well as a decrease in the risk of developing coronary heart diseases, hypertension, diabetes, obesity, and gastrointestinal disorders [3,4]. Besides health benefits, fibers possess some technological and functional attributes that allow them to be used as thickeners, oil texturizers, and moisture retention agents during the preparation of different types of products, including yogurts, ice creams, sauces, dressings, beverages, meat, and bakery products [5,6,7,8]. The apple fibers that were chosen for this experiment in addition to cellulose, hemicellulose, and pectin are also a rich source of bioactive compounds [9]. Some progress has already been made in investigations concerning the interactions of dietary fibers and phenolics and volatiles. Hydroxytyrosol and 3,4-dihydroxyphenylglycol, which are phenolics with important biological activity, were complexed with strawberry dietary fibers in order to formulate effective functional food ingredients that could potentially promote intestinal health [10]. Next to strawberry dietary fibers, these phenolics were also complexed with pectin, with the aim of formulating complexes that could potentially be used for colon targeting [11,12]. Pectin and cellulose as cell wall models can be used as carriers of anthocyanins and phenolic acids since they can bind those phenolics [13,14]. Not all phenolics equally bind to dietary fibers. For example, procyanidins bound to components of the apple cell wall, while hydroxycinnamic acids and epicatechin exhibited the opposite trend [15]. The polysaccharides of the apple cell wall affected the antioxidant activity of quercetin [16], while fiber from onions did not [17]. Cellulose can not only be used as an efficient carrier of raspberry phenolics, but also of raspberry volatiles; thus, the obtained complexes can be used as colorants and flavorings in the food industry [18,19]. In our study, we selected blackberries as a source of phenolic and volatile compounds. Blackberries are known as good sources of phenolics with high antioxidant potential, which are responsible for diverse health benefits [20,21]. In addition to their appealing color, blackberries are also known for their pleasant flavor [22,23].

Even with the existence of many different encapsulation techniques [24,25], and although higher unit costs for freeze-drying are a disadvantage, this technique is still widely used to obtain high-value food products, especially when the goal is to have higher polyphenol retention [26,27]. The conditions of the selected encapsulation technique, the chemical and physical properties of the carriers, and active ingredients play a major role in encapsulation efficiency [24]. In order to improve the encapsulation of the phenolics and volatiles of blackberry juice with apple fibers by freeze-drying, two disaccharides were selected, namely sucrose and trehalose. As far as we know, this combination of carriers has not been studied. Sucrose is a commonly used saccharide in fruit product formulations, while the use of trehalose in formulations of fruit products is increasing in frequency. The positive influence of trehalose on volatiles and phenolics in different freeze-dried fruit products has been proven in several studies [28,29,30,31,32,33,34,35,36,37,38,39], therefore we wanted to determine whether a similar effect could be achieved in combination with apple fibers. An additional benefit of trehalose is its slow digestion, which results in a lower glycemic index, with a lower insulin release compared to sucrose [40]. Likewise, trehalose has substantially lower cariogenic potential compared to sucrose, and it does not have laxative effects similar to the other low-cariogenic bulk sweeteners [41].

The results of our previous research [42] showed that apple fibers could be good carriers of blackberry polyphenols and volatiles. The aim of this study was to evaluate the effect of the addition of disaccharides on the encapsulation of the phenolic and volatile compounds of blackberry juice with apple fiber. In order to investigate whether disaccharides can improve the encapsulation of the mentioned compounds with apple fiber, sucrose and trehalose were used in fiber:disaccharides ratios of 1:0.5, 1:1, and 1:2. All formulated microparticles were evaluated for total phenolics, proanthocyanidins, individual phenolic content, antioxidant activity, color parameters, and flavor profile.

## 2. Materials and Methods

### 2.1. Chemicals

Apple fibers in powder form was obtained from Biesterfeld AG (Zagreb, Croatia). 2,2-diphenyl-1-picrylhydrazyl (DPPH), 2,2′-azino-bis(3-ethylbenzothiazoline-6-sulfonic acid) diammonium salt (ABTS), trolox, 4-(dimethylamino)-cinnamaldehyde (DMAC), chlorogenic, caffeic, p-coumaric, gallic and ellagic acids, rutin, quercetin, phloretin, phlorizin, and myrtenol were purchased from Sigma-Aldrich (St. Louis, MO, USA). Potassium persulfate, Folin-Chiocalteu reagent, and sodium carbonate were obtained from Kemika (Zagreb, Croatia). Neocuproine, 2,4,6-tri(2-pyridyl)-s-triazine (TPTZ), and copper (II) chloride were purchased from Acros Organics (Geel, Belgium). Methanol (HPLC grade) was acquired from J.T. Baker (Deventer, The Netherlands), and orthophosphoric acid (HPLC grade > 85%) was obtained from Fisher Scientific (Loughborough, UK). Iron (III) chloride hexahydrate, sodium acetate, ethanol, ammonium acetate, and starch were bought from Gram-mol (Zagreb, Croatia). Cyanidin 3-glucoside was obtained from Extrasynthese (Genay, France), and 3,5-dinitrosalicylic acid was from Alfa Aesar (Kandel Germany). Sucrose was obtained from Fluka (Buchs, Switzerland). Trehalose was a donation from Hayashibara Company (Nagase group, Okayama, Japan).

### 2.2. Formulation of Microparticles

Blackberry juice was prepared by pressing the berries through the press and additionally filtrating them through cheesecloth to remove solids in order to obtain clear juice. Subsequently, the obtained juice was heated at 90 °C for 3 min to inactivate enzymes. Complexation was performed by mixing selected ingredients: apple fibers and blackberry juice for the control sample, and apple fibers, disaccharides, and blackberry juice for the microparticles, with the addition of disaccharides. For the formulation of the control sample, apple fibers (3 g) were added to blackberry juice (50 mL). The obtained mixture was homogenized by stirring on a magnetic stirrer for 20 min. Microparticles with the added disaccharides (sucrose or trehalose) were prepared by the addition of apple fibers and disaccharides into blackberry juice, followed by the previously described homogenization process. For these microparticles, the fiber:disaccharides ratios were 1:0.5, 1:1, and 1:2. Prepared homogenized mixtures of the control sample and the microparticles with the added disaccharides were transferred to cuvettes and centrifuged for 15 min (4000 rpm). Phenolics and volatiles from blackberry juice adsorbed onto the apple fibers or apple fibers/disaccharides represented the wet precipitate, which was further used to obtain dried microparticles. The fluid part that contained unadsorbed blackberry phenolics and volatiles was discarded. Freeze-drying was performed in a laboratory freeze-dryer (Christ Freeze Dryer, Alpha 1–4, Osterode am Harz, Germany). Firstly, before freeze-drying, wet precipitates were frozen (−18 °C). Conditions for freeze-drying were set as follows: freezing temperature was −55 °C, the temperature of sublimation was from −35 °C to 0 °C, and the vacuum level was 0.220 mbar. Temperatures of the isothermal desorption varied from 0 °C to 21 °C under the vacuum of 0.060 mbar. The complete process of freeze-drying lasted for 12 h.

### 2.3. Extraction of Microparticles

Extraction of microparticles was necessary in order to evaluate the total phenolics, proanthocyanidins, individual phenolics, and antioxidant activity. A total of 0.2 g of microparticles was weighted, and 10 mL of acidified methanol (HCl:methanol ratio was 1:99) was added. Obtained mixtures were mixed and homogenized on a multi-speed vortex. These homogenized mixtures were left in the dark for 24 h and then filtered. The resulting extracts were used for the mentioned analyses.

### 2.4. Evaluation of the Total Phenolics

The total phenolic content in the samples was determined by the Folin–Ciocalteu method [43]. In brief, in a glass tube, 0.2 mL of the extract and 1.8 mL of deionized water were added and mixed. To this mixture, 10 mL (1:10) of the Folin–Ciocalteu reagent and 8 mL of sodium carbonate (7.5%) were added and mixed. The complete mixture was incubated at room temperature in the dark for 2 h, and then the absorbance was measured at 765 nm on a UV/Vis spectrophotometer (Cary 60 UV-Vis, Agilent Technologies, Santa Clara, CA, USA). The measurements were performed in triplicate, and the results were expressed as g of gallic acid equivalents per 100 g of microparticles (g GAE/100 g).

### 2.5. Evaluation of Total Proanthocyanidins

Total proanthocyanidin content was determined by the application of the colorimetric DMAC method [44]. Briefly, in a glass tube, an aliquot of the extract and a 4-(dimethylamino)-cinnamaldehyde solution were added and mixed. The complete mixture was incubated for 30 min, and then the absorbance was measured at 640 nm. The measurements were performed in triplicate, and the results were expressed as mg of procyanidin B2 equivalents per 100 g of microparticles (mg PB2E/100 g).

### 2.6. Evaluation of Antioxidant Activity

The ABTS assay followed the method of Arnao et al. [45], with some modifications. Briefly, 0.1 mL of extract was mixed with 3 mL of ABTS reagent and then left in the dark. The antioxidant activity of the samples was also determined by the radical scavenging activity method using 2,2-diphenyl-1-picrylhydrazyl (DPPH) [46]. Cupric reducing antioxidant capacity assay was carried out according to the method of Apak et al. [47]. Moreover, the antioxidant capacity of the samples was determined according to the Benzie and Strain method [48], with some modifications. For each method for blank, the extract was replaced with water, and all measurements were performed in triplicate. Antioxidant activities evaluated by ABTS, DPPH, FRAP, and CUPRAC methods were calculated from the calibration curve, with Trolox as the standard and expressed as µmol TE/100 g.

### 2.7. Identification and Quantification of Phenolic Compounds

Extracts were filtered through 0.2 µm PTFE membranes and evaluated using an HPLC (high-performance liquid chromatography) system 1260 Infinity II (Agilent technology, Santa Clara, CA, USA). The apparatus was equipped with a quaternary pump, a vial sampler, DAD detector, and a Poroshell 120 EC C-18 column (4.6 × 100 mm, 2.7 µm). The method used to evaluate the phenolic compounds was previously described in a study by Ivić et al. [49]. Calibration curves were obtained for cyanidin-3-glucoside, quercetin, chlorogenic acid, ellagic acid, rutin, caffeic acid, p-coumaric acid, gallic acid, phloretin, and phlorizin hydrate. Cyanidin-3-dioxalylglucoside and two derivates of hydroxycinnamic acids were tentatively identified by comparing their peak spectrum with standards and literature data, and were then quantified using cyanidin-3-glucoside and chlorogenic acid, respectively.

### 2.8. Color Measurement and Color Change

Chromometer Minolta CR-400 (Minolta; Osaka, Japan) was used for the evaluation of the color parameters of the formulated microparticles by Lab system. The measured color parameters were as follows: L* (lightness; 0 is black and 100 is white), a* (redness (+) and greenness (−)), b* (yellowness (+) and blueness (−)), C* (the color saturation value-chroma), and °h (the hue angle). The L*, a*, and b* values were used for the calculation of total color change, ΔE.

### 2.9. Evaluation of Volatiles

Volatiles from the formulated microparticles were extracted by solid-phase microextraction (SPME). A total of 0.3 g of the sample, 4.7 g of water, and 1 g of NaCl were weighed into a 10 mL glass vial. For the extraction of volatiles, SPME fiber coated with a divinylbenzene/carboxen/polydimethylsiloxane (DVB/CAR/PDMS) sorbent (50/30 µm, StableFlex™, Supelco, Bellefonte, PA, USA) was used. The method was described by Vukoja et al. [18]. Compounds were confirmed by matching their mass spectra with the National Institute of Standards and Technology mass spectral database (NIST, East Amwell Township, NJ, USA) and through their retention time (RT) and retention index (RI). Two repetitions were made for each sample. Quantification was conducted with myrtenol as an internal standard, and results were expressed as µg/kg.

### 2.10. Fourier Transform Infrared with Attenuated Total Reflection (FTIR-ATR) Spectroscopy Analysis

The FTIR-ATR (Cary 630, Agilent, Santa Clara, CA, USA) technique was used to obtain the infrared (IR) spectra of the microparticles. The observed IR spectra are the absorbance of different microparticles versus the wavenumber range, from 4000 cm^−1^ to 600 cm^−1^.

### 2.11. Statistical Analysis

A comparison of the formulated microparticles was conducted by analysis of variance (ANOVA) and Fisher’s least significant difference (LSD), with the significance defined at *p* < 0.05. Additionally, principal component analysis (PCA) on the volatile compounds was conducted. The software program STATISTICA 13.1 (StatSoft Inc., Tulsa, OK, USA) was used for the statistical analyses. Results were presented as the mean values ± standard deviation.

## 3. Results

### 3.1. Phenolic Compounds, Antioxidant Activities, and Color of Formulated Microparticles

**Figure 1:**
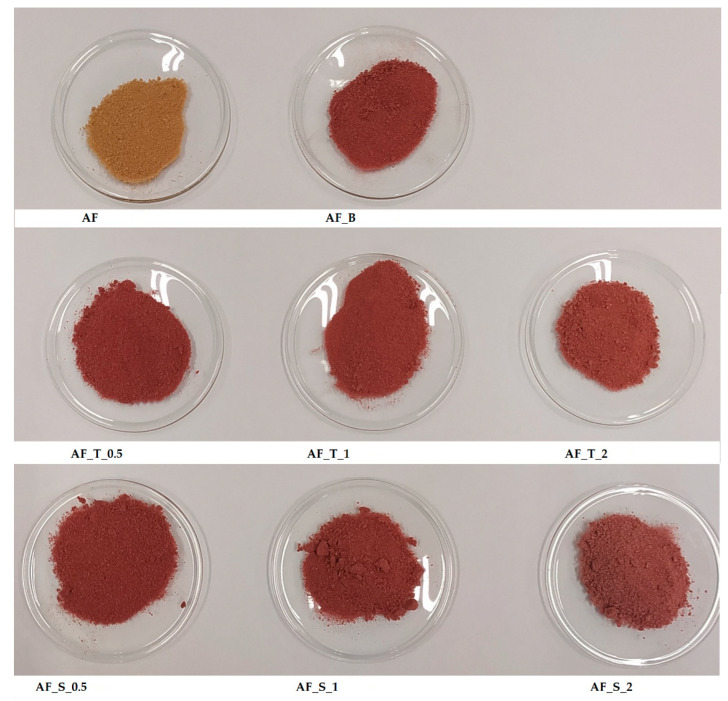
Presentation of formulated microparticles. AF_B—apple fiber/blackberry microparticles (control sample); AF—apple fiber; S—sucrose; T—trehalose; 0.5—ratio of apple fibers:disaccharide (1:0.5); 1—ratio of apple fibers:disaccharide (1:1); 2—ratio of apple fibers:disaccharide (1:2).

Apple fibers, apple fiber/blackberry juice microparticles as control sample, and microparticles with the added disaccharides are presented in Figure 1.

Results of the total phenolics (TP) and proanthocyanidin (PA) content of the formulated microparticles are presented in Table 1. The highest TP content had microparticles that were formulated only with apple fiber and blackberry juice (1.35 GAE g/100 g). The addition of disaccharides during the formulation of the microparticles caused a decrease of TP, i.e., adsorption of phenolics onto the apple fiber was lower in comparison to the apple fiber without the added disaccharides. An increase of added disaccharides had a negative effect on the adsorption of phenolics, and there was no change among microparticles formulated with sucrose and trehalose. Considering only proanthocyanidins, a different trend was observed as compared to total phenolics. The microparticles prepared only with apple fiber had the highest PA content (47.81 mg/100 g). However, unlike for TP, the difference between disaccharide types was observed. The addition of trehalose during formulation had a more positive impact on the adsorption of PA onto the apple fiber than the addition of sucrose. When trehalose was applied during the formulation of the microparticles, higher PA content was adsorbed onto the apple fiber, and with the increase in the amount of trehalose, a decrease of PA content occurred (42.99 mg/100 g, 40.67 mg/100 g, and 39.88 mg/100 g). The same tendency, i.e., a decrease in the adsorption of proanthocyanidins with the increase of sucrose occurred, but PA content was significantly lower (26.42 mg/100 g, 21.41 mg/100 g, and 12.98 mg/100 g).

Individual phenolic compounds of blackberry juice and apple fiber are presented in Table 2. The type of disaccharides and their ratio to apple fiber affected the adsorption of individual phenolic compounds (Table 3). Blackberry juice contained quercetin, rutin, cyanidin-3-glucoside, cyanidin-3-dioxalylglucoside, ellagic acid, caffeic acid, chlorogenic acid, p-coumaric acid, and gallic acid, while in microparticles, anthocyanins, quercetin, ellagic acid, and chlorogenic acid were determined with the two derivatives of hydrocinnamic acids—phloretin and phlorizin hydrate; these two derivatives originated from apple fibers. Cyanidin-3-glucoside was determined in the highest amount in the apple fiber/blackberry microparticles (151.97 mg/100 g). With the increased addition of disaccharides, lower amounts of this anthocyanin was detected, which means that the addition of disaccharides during the formulation of microparticles negatively affected the adsorption of this compound onto the apple fiber. Regardless of the disaccharide type, with the increased amounts of added disaccharides, a decrease in cyanidin-3-glucoside content was observed. When the fiber:disaccharides ratios were 1:0.5 and 1:2, higher anthocyanin content was observed in the trehalose microparticles, while the opposite results were observed for the third type of microparticles. The highest amount of cyanidin-3-dioxalylglucoside was also observed in apple fiber/blackberry microparticles (36.71 mg/100 g). Similarly, for cyanidin-3-glucoside, the increased addition of disaccharides lowered the amount of this anthocyanin. Similar results were also obtained for quercetin. The highest amount of this phenolic (92.96 mg/100 g) was determined in the apple fiber/blackberry microparticles. Other microparticles had lower quercetin content. In this case, when the fiber:disaccharide ratios were 1:0.5 and 1:2, higher quercetin content was observed in the trehalose microparticles, while there was no difference for the third type of microparticles. Ellagic acid was also detected in the highest amount in the apple fiber/blackberry microparticles (28.95 mg/100 g). Regarding microparticles with disaccharides, the same trend as for quercetin was observed, thus the trehalose microparticles had a slightly higher content of this phenolic acid. Chlorogenic acid as well as two derivatives of hydroxycinnamic acid were also determined in the microparticles. All phenolic acids were also determined in the highest amount in the apple fiber/blackberry microparticles, but the differences between those microparticles and the microparticles with disaccharides were not so high.

Antioxidant activities of the formulated microparticles were evaluated by the application of the DPPH, ABTS, FRAP, and CUPRAC methods (Table 1). For all four methods, it was observed that apple fiber microparticles had the highest antioxidant activities, while all the other microparticles with added disaccharides had lower values. In fact, by comparing the results of antioxidant activities with the results of phenolics, it can be concluded that antioxidant activity followed the same trend. Higher values for microparticles with trehalose addition were obtained with the FRAP and especially CUPRAC methods, which can be correlated with the higher amount of proanthocyanidins adsorbed onto those microparticles.

Furthermore, the L*, a*, and b* color parameters were recorded in order to establish whether disaccharides had influenced the color of formulated microparticles (Table 4). The L* value of disaccharide microparticles was higher than that of microparticles formulated only with apple fiber. In addition, it was observed that with the increase in the amount of disaccharides, the L* values increased. When the fiber:disaccharides ratios were 1:0.5 and 1:1, the L* values were equal regardless of the disaccharide type. The microparticles with the highest amount of trehalose had a higher L* value than the corresponding sucrose microparticles. The a* and b* values of the disaccharide microparticles were higher than those for microparticles formulated only with apple fiber. Slightly higher values were observed for microparticles formulated with sucrose. Based on L*, a*, and b*, the color change (ΔE) was calculated for the microparticles with disaccharides relative to the apple fiber microparticles. The color change increased with the increase in disaccharides, and the highest difference between microparticles was observed when they were added in the highest amount. The °h and C* values were also determined. Slight changes in these parameters were observed in microparticles with added disaccharides as compared to the apple fiber microparticles.

### 3.2. Flavor Profile of Formulated Microparticles

Seventeen volatiles were determined in the microparticles, while 16 were identified in blackberry juice (minus hexanal in compared microparticles) and 9 in apple fiber (minus linalool oxide, guaiacol, phenethyl alcohol, menthol, nerol, and eugenol as compared to microparticles). Apple fiber/blackberry microparticles were used as a control for the comparison of volatiles in microparticles with disaccharides, i.e., the effect of disaccharides on the adsorption of volatiles (Table 5 and Table 6). Disaccharide type and their ratio to the fiber during formulation had an effect on the adsorption of volatile compounds. Two aldehydes were determined—hexanal and heptanal. Both of them were determined in higher amounts in the microparticles with added disaccharides than on apple fiber. With the increase in disaccharides, an increase in their amount in microparticles occurred, with trehalose having a more positive effect. Phenethyl and perillyl alcohols had a different trend than aldehydes. The amount of phenethyl alcohol was the highest in the apple fiber microparticles. For the other microparticles, it was observed that with the increase of disaccharides, a decrease in the adsorption of this volatile occurred. For perillyl alcohol, it was determined that microparticles with the lowest amount of disaccharides had higher content than the apple fiber microparticles. Terpenes were the most abundant volatiles in the microparticles. D-limonene was determined in the highest amount in all microparticles. Equal amounts of this terpene were determined in the apple fiber microparticles and the microparticles with sucrose (fiber:disaccharide ratios 1:0.5 and 1:1), while the third sucrose microparticles had the lowest amount of D-limonene. Trehalose microparticles showed different behavior, i.e., the highest amount of this terpene was determined in the microparticles prepared with a fiber:disaccharide ratio of 1:2. At lower amounts of trehalose, absorption of this volatile decreased. Citronellal and linalool had similar behavior. Microparticles with disaccharides (fiber:disaccharide ratios 1:1 and 1:2) had lower contents of those terpenes compared to apple fiber microparticles. A different effect was observed for microparticles with fiber:disaccharide ratio of 1:0.5. Microparticles with sucrose had an equal amount as apple fiber microparticles, while trehalose microparticles had the highest content of these terpenes. For guaiacol, it was determined that all trehalose microparticles had a higher amount of this volatile than the one with just apple fiber, while for sucrose microparticles, this trend was observed only when its lowest amount was used for the formulation. Menthol and β-damascenone had similar behavior. The highest amount of these terpenes was determined in the apple fiber microparticles. For disaccharide microparticles, a decreased adsorption of those terpenes occurred with the increase in disaccharides. Both microparticles with the fiber:disaccharide ratio of 1:0.5 had higher amounts of eugenol and citral than the apple fiber microparticles. For nerol, the highest content was determined in the apple fiber microparticles, α-ionone was the highest in microparticles with disaccharides (fiber:disaccharide ratio of 1:0.5), while microparticles with the fiber:disaccharide ratios of 1:1 and 1:2 had equal or lower amounts of this terpene than apple fiber, respectively. For γ-ionone, it was determined that only microparticles with trehalose (fiber:disaccharide ratio of 1:0.5) had higher content than apple fiber, while other microparticles were lower. Similarly, with the increase in disaccharides, a decrease of this terpene occurred. All disaccharide microparticles had lower β-ionone content than the apple fiber microparticles, but a positive effect of trehalose was noted.

The flavor profile of any product depends on the amount of volatiles, but also on their odor thresholds. To further elucidate the contribution of each volatile to the overall flavor, odor activity values (OAVs) were calculated. OAVs were calculated as the ratio of the volatile concentration to the threshold [22]. A summary of OAVs of all the samples based on published odor thresholds is presented in Table 7. In blackberry juice, linalool, guaiacol, eugenol, β-damascenone, α-ionone, γ-ionone, and β-ionone had OAV values over 1, which indicates their importance to the flavor of blackberry juice. Especially high OAVs (over 10) were calculated for γ-ionone, β-ionone, and α-ionone (29.71, 25.80, and 21.83, respectively). For apple fiber, OAVs between 1 and 10 were calculated for hexanal, heptanal, D-limonene, and α-ionone. Volatiles with OAVs over 10 were linalool, γ-ionone, and β-ionone (13.49, 30.57, and 54.60, respectively). Microparticles contained flavors from both sources. Comparing all microparticles, OAVs between 1 and 10 were calculated for hexanal, citronellal, and α-ionone, while D-limonene and linalool had OAVs slightly over 10, and γ-ionone and β-ionone had very high OAVs. Heptanal was the only volatile that was not important for the flavor of the apple fiber/blackberry microparticles, but it was important for other microparticles with added disaccharides (OAVs were between 1 and 3). Comparing microparticles, especially high differences between the apple fiber/blackberry microparticles and the microparticles with disaccharides were detected for γ-ionone and β-ionone. Even though several volatiles (guaiacol, eugenol, and β-damascenone) had high OAVs in blackberry juice, they were not important for the flavor of the microparticles.

**Figure 2:**
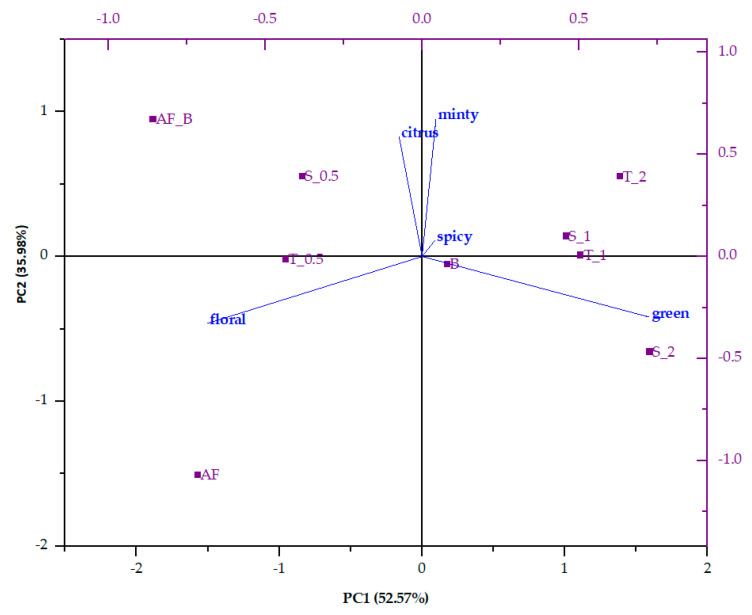
Principal component analysis (PCA) biplot of volatile compounds in blackberry juice, apple fiber, and formulated microparticles. B—blackberry juice; AF—apple fiber; AF_B—apple fibers/blackberry microparticles; S—sucrose; T—trehalose; 0.5—ratio of apple fibers:disaccharide (1:0.5); 1—ratio of apple fibers:disaccharide (1:1); 2—ratio of apple fibers:disaccharide (1:2).

Volatiles of all the samples were grouped according to their flavor descriptors, and the contributions of each flavor note to the overall flavor profile were calculated. Results were used for PCA analysis (Figure 2). PC1 accounted for 52.57% of the total variance, and PC2 for 35.98%. For all samples, dominant flavor notes were floral, green, and citrus. From the biplot, it can be observed that apple fiber had quite a different flavor profile; it was missing spicy and minty flavor notes but had a pronounced floral note, followed by green and citrus ones (approximately equal contributions). Blackberry juice was characterized mostly by a floral flavor note, followed by a green note; the citrus note had the lowest contribution to the overall flavor. The microparticles from blackberry juice and apple fiber were different since their flavor was a mixture of both volatiles that originated from blackberry juice and apple fiber. Apple fiber/blackberry microparticles was characterized mostly by floral and citrus flavor notes. Both microparticles formulated by the addition of sucrose and trehalose (fiber:disaccharide ratio of 1:0.5) were similar in terms of their flavor profile in order to the control sample, i.e., they were characterized mostly by floral and citrus flavor notes. With the increase in added disaccharides, the flavor profile changed, and for those microparticles, green flavor notes prevailed. Interestingly, both microparticles were similar when the fiber:disaccharide ratio was 1:1. The green note was the dominant one, while floral and citrus notes had an equal contribution to the overall flavor. A change in the fiber:disaccharide ratio to 1:2 created a difference between the microparticles with trehalose and sucrose. Green and citrus flavor notes were the dominant ones for microparticles with trehalose, while in the microparticles with sucrose, only the green flavor note was dominant; the other two flavor notes were in equal ratio.

### 3.3. IR Spectra

**Figure 3:**
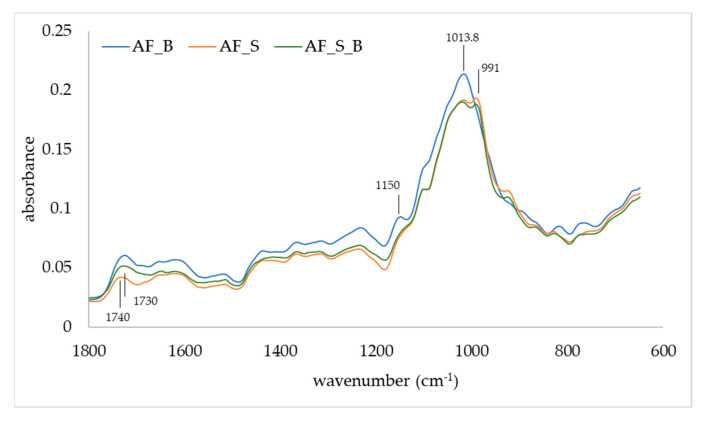
IR spectra of apple fiber/blackberry (AF_B), apple fiber/sucrose (AF_S), and apple fiber/sucrose/blackberry (AF_S_B) microparticles.

**Figure 4:**
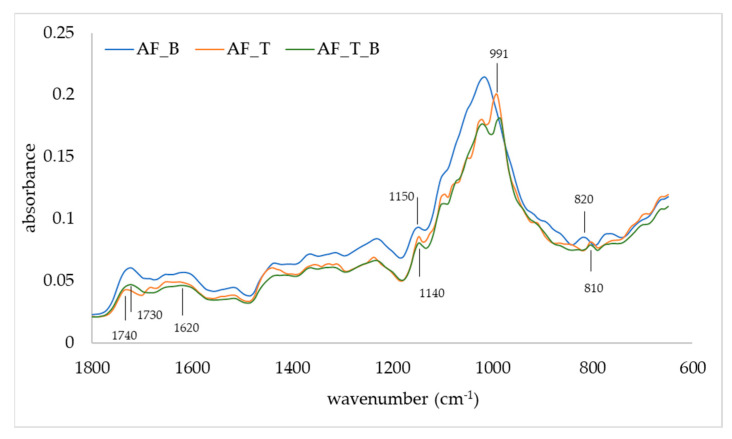
IR spectra of apple fiber/blackberry (AF_B), apple fiber/trehalose (AF_T), and apple fiber/trehalose/blackberry (AF_T_B) microparticles.

The FTIR-ATR was used to observe changes in the IR spectra of apple fibers upon adsorption of blackberry compounds and disaccharides (Figure 3 and Figure 4). Apple fiber/blackberry microparticles had a band at 1013.8 cm^−1^, while on apple fiber/disaccharide and apple fiber/disaccharide/blackberry microparticles, this band was wider, with the additional band at 991 cm^−1^, which is a consequence of the binding of disaccharides onto the apple fiber. The band at 1013.8 cm^−1^ is assigned to ring stretching vibrations mixed strongly with CH in-plane bending, while the band at 991 cm^−1^ is assigned to C-O [43]. The band at 1150 cm^−1^ (assigned to C-O stretching mode of the carbohydrates) disappeared due to the binding of sucrose, while the binding of trehalose caused a shift of this band to 1140 cm^−1^. Additional change in the IR spectra was observed upon the binding of sucrose in the region 950–750 cm^−1^. Changes in the IR spectra were also observed upon adsorption of blackberry compounds, in both the apple fiber/blackberry and apple fiber/disaccharide/blackberry microparticles. These changes included the shift of a band at 1740 cm^−1^ (which is assigned to C=O) to 1730 cm^−1^, and its intensity in comparison to 1620 cm^−1^ was higher than in apple fiber/disaccharide microparticles. Additional change upon adsorption of the blackberry compounds was observed in the trehalose microparticles, i.e., the band at 810 cm^−1^ (assigned to ring CH deformation) shifted to 820 cm^−1^ [50].

## 4. Discussion

Previous studies showed that the efficiency of the encapsulation of phenolics by carriers depended on the type, amount, and properties of phenolics as well as the carriers [19,51,52,53]. It was determined that raspberry phenolics effectively bound to cellulose, and the binding depended on the amount of cellulose and the time required for complexation [19]. In the study on the selective binding of ferulic acid, cyanidin-3-glucoside, and catechin onto cellulose-based composites and apple cell walls, it was observed that cellulose was the dominant binding component for catechin, while xyloglucan and arabinoxylan did not contribute to the adsorption of this compound in the presence of cellulose. The other two compounds bound to selected materials with different affinities. Both electrostatic forces and plant cell wall microstructure were very important for binding; thus, the negatively-charged pectin on cell walls exhibited the most extensive binding to positively charged cyanidin-3-glucoside, while its binding with negatively-charged ferulic acid was the least effective [53]. It was determined that the interaction between different phenolics (such as catechin, ferulic acid, chlorogenic acid, gallic acid, and cyanidin-3-glucoside) and cellulose occurred spontaneously, within 1 min, and rapidly increased over 30 min [54]. In addition, the authors determined that chlorogenic acid had different behavior as compared to other investigated phenolics. While all other phenolics bound similarly on a molar basis, the binding of chlorogenic acid was lower [54]. It was postulated by Liu et al. [55] that the initial binding of phenolics and cellulose occurred due to the adsorption of phenolics onto the binding sites of the cellulose surface, which resulted from the presence of labile hydroxyl groups; after that, non-covalent binding, i.e., hydrogen bonding and hydrophobic interactions could occur [55]. This could also be the mechanism of interaction between blackberry juice phenolics and apple fiber and apple fiber/disaccharides. Very important factors for non-covalent binding were also phenolic rings, i.e., their number and their conformational flexibility [56]. In our study, we had mixtures of phenolics, and in addition to apple fiber (consisting of cellulose, hemicellulose, and pectin), we also had disaccharides, which played an important part in the binding of phenolics to apple fiber. It is possible that when interactions between apple fiber and disaccharides were formed, there were no remaining binding sites for phenolics since it was observed that the apple fiber/blackberry microparticles had the highest phenolic content and antioxidant activities. On the other hand, there were differences between the sucrose and trehalose containing microparticles and their amounts, which also proves that the type of disaccharides and their properties can affect the binding of phenolics. The unique structure of trehalose enables trehalose to interact with both hydrophilic and hydrophobic molecules [57]; thus, this property could be the reason for the differences between the sucrose and trehalose microparticles. The differences caused by disaccharide types in fruit samples were proven in several studies [33,35,36,37,39,58], and in most cases, trehalose had a more positive effect on phenolics and antioxidant activity.

As was already mentioned in the introduction, trehalose had a positive influence on volatiles in different freeze-dried fruit products [28,29,30,31,32,34], and we also observed that trend in our type of products. As it was observed for phenolic compounds as well as for volatiles, we can conclude that the type, amount, and properties of both volatiles and carriers played important roles in the retention of volatile compounds. Additionally, competition between components of binding sites has to be accounted for since it has been proven that this could be a significant factor in the retention of volatiles, especially when they are added as a mixture of compounds [31,34,59], as is the case in this study. Even though trehalose and sucrose are chemical isomers, from our results on phenolics and volatiles, they had different influences on those compounds since they differ in their behavior. The formation of stable intramolecular complexes between trehalose and unsaturated compounds [57,60,61] is also a possible explanation for the positive effect of trehalose on phenolics and volatiles. Volatiles are characterized by their diffusion. Sugars have different diffusion coefficients in water and can change the diffusion coefficient of water [62]; therefore, through the modification of the diffusion coefficient of volatiles, they can influence their retention in the fruit product matrix. We can thus conclude that disaccharides had an influence on water dynamics during complexation, resulting in the effect of sugars on volatile and phenolic compounds.

## 5. Conclusions

Based on the results of this study, it can be concluded that apple fibers are good encapsulating material for blackberry juice phenolic and volatile compounds. The enhancement of the adsorption of those compounds depended on the type and amount of disaccharides. The addition of sucrose and trehalose decreased the adsorption of phenolics onto the apple fiber. When comparing the influence of applied disaccharides, it was observed that trehalose had a higher positive effect on adsorption than sucrose. Generally, the adsorption of volatiles was higher with the application of disaccharides in comparison only to apple fibers. Comparing sucrose and trehalose, once again, trehalose had a more positive effect than sucrose. These results show that the formulation of encapsulates, i.e., the selection of carriers, had an important role in the final quality of the microparticles. These microparticles can be used for the enrichment of conventional food products with phenolics and volatiles, but also for the development of novel foods, especially functional foods.

## Figures and Tables

**Table 1:** Total phenolics (TP), proanthocyanidins (PA), and antioxidant activity of formulated microparticles.

Samples	TP	PA	DPPH	ABTS	FRAP	CUPRAC
AF_B	1.35 ± 0.01 ^a^	47.81 ± 0.12 ^a^	53.18 ± 0.02 ^a^	65.65 ± 0.32 ^a^	9.13 ± 0.66 ^a^	555.39 ± 4.24 ^a^
AF_S_0.5	1.29 ± 0.02 ^b^	26.42 ± 0.36 ^e^	48.05 ± 1.01 ^c^	55.98 ± 0.57 ^b^	7.55 ± 0.78 ^b^	494.75 ± 7.29 ^c^
AF_S_1	1.15 ± 0.02 ^d^	21.41 ± 0.18 ^f^	47.80 ± 0.35 ^c^	46.95 ± 0.98 ^d^	7.04 ± 0.53 ^c^	451.29 ± 7.21 ^d^
AF_S_2	0.95 ± 0.02 ^f^	12.98 ± 0.59 ^g^	42.16 ± 0.51 ^e^	41.74 ± 0.74 ^e^	5.98 ± 0.41 ^d^	378.74 ± 6.85 ^e^
AF_T_0.5	1.25 ± 0.01 ^b^	42.99 ± 0.60 ^b^	51.23 ± 0.01 ^b^	54.22 ± 0.92 ^b^	8.06 ± 0.58 ^a^	551.79 ± 7.77 ^a^
AF_T_1	1.15 ± 0.01 ^c^	40.67 ± 0.19 ^c^	47.93 ± 1.42 ^c,d^	51.51 ± 0.35 ^c^	7.65 ± 0.69 ^b^	506.46 ± 0.38 ^b^
AF_T_2	1.02 ± 0.02 ^d^	39.88 ± 0.07 ^d^	45.55 ± 0.72 ^d^	45.98 ± 0.25 ^d^	6.41 ± 0.43 ^c^	449.94 ± 1.42 ^d^

AF_B—apple fiber/blackberry microparticles (control sample); AF—apple fiber; S—sucrose; T—trehalose; 0.5—ratio of apple fibers:disaccharide (1:0.5); 1—ratio of apple fibers:disaccharide (1:1); 2—ratio of apple fibers:disaccharide (1:2). TP—expressed as g GAE/100 g; PA—expressed as mg PB2/100 g; antioxidant activity—expressed as µmol TE/100 g. Values in the same column marked with different superscripts (a–g) are statistically different.

**Table 2:** Individual phenolic compounds (mg/100 g) of blackberry juice and apple fiber.

Blackberry Juice		Apple Fiber	
Cyanidin 3-glucoside	339.8 ± 0.40	Phloretin	17.64 ± 0.48
Cyanidin 3-dioxalylglucoside	118.8 ± 0.03	Phlorizin hydrate	78.21 ± 0.60
Ellagic acid	27.35 ± 0.00	Chlorogenic acid	51.64 ± 2.70
Caffeic acid	3.8 ± 0.00	HC-1	17.36 ± 0.13
Chlorogenic acid	31.55 ± 0.03	HC-2	15.72 ± 0.02
p-Coumaric acid	41.1 ± 0.00	Quercetin	130.60 ± 5.38
Gallic acid	36.3 ± 0.01	Rutin	9.54 ± 1.14
Quercetin	22.7 ± 0.05		
Rutin	3.7 ± 0.00		

HC-1 and HC-2—two derivatives of hydroxycinnamic acid.

**Table 3:** Individual phenolic content (mg/100 g) in formulated microparticles.

	AF_B	AF_S_0.5	AF_S_1	AF_S_2	AF_T_0.5	AF_T_1	AF_T_2
C-3-G	151.97 ± 0.11 ^a^	138.74 ± 0.42 ^c^	127.01 ± 2.88 ^d^	100.28 ± 0.28 ^g^	142.56 ± 1.06 ^b^	120.17 ± 1.28 ^e^	111.97 ± 4.39 ^f^
C-3-DG	36.71 ± 0.46 ^a^	32.58 ± 0.31 ^b^	30.61 ± 0.32 ^c^	24.90 ± 0.38 ^f^	32.20 ± 0.07 ^b^	27.98 ± 0.36 ^d^	26.20 ± 0.54 ^e^
Q	92.96 ± 1.14 ^a^	81.72 ± 0.36 ^c^	75.99 ± 1.02 ^d^	62.93 ± 0.12 ^f^	83.69 ± 0.00 ^b^	74.64 ± 1.19 ^d^	68.27 ± 1.44 ^e^
EA	28.95 ± 0.01 ^a^	24.35 ± 0.09 ^c^	23.43 ± 0.47 ^d^	20.20 ± 0.09 ^e^	26.32 ± 0.26 ^b^	22.89 ± 0.71 ^d^	22.77 ± 0.65 ^d^
ChA	23.63 ± 0.28 ^a^	21.58 ± 0.10 ^c^	21.67 ± 0.20 ^c^	19.25 ± 0.01 ^d^	22.32 ± 0.15 ^b^	20.89 ± 0.25 ^c^	19.66 ± 0.32 ^d^
HC-1	21.24 ± 0.04 ^a^	19.59 ± 0.00 ^c^	19.91 ± 0.12 ^c^	18.08 ± 0.02 ^e^	20.65 ± 0.07 ^b^	19.78 ± 0.07 ^c^	19.16 ± 0.22 ^d^
HC-2	17.82 ± 0.04 ^a^	16.61 ± 0.00 ^d^	17.16 ± 0.07 ^b,c^	15.83 ± 0.02 ^e^	17.40 ± 0.00 ^b^	16.93 ± 0.09 ^c^	16.48 ± 0.09 ^d^
P	16.98 ± 0.03 ^e^	14.45 ± 0.01 ^c^	10.89 ± 0.27 ^b^	9.09 ± 0.01 ^a^	16.47 ± 0.09 ^e^	15.39 ± 0.38 ^d^	14.84 ± 0.61 ^c^
Ph	22.47 ± 0.07 ^e^	20.33 ± 0.49 ^d^	15.45 ± 0.07 ^c^	13.39 ± 0.37 ^b^	15.83 ± 0.08 ^c^	13.45 ± 0.45 ^b^	11.21 ± 0.28 ^a^

AF_B—apple fiber/blackberry microparticles (control sample); AF—apple fiber; S—sucrose; T—trehalose; 0.5—ratio of apple fibers:disaccharide (1:0.5); 1—ratio of apple fibers:disaccharide (1:1); 2—ratio of apple fibers:disaccharide (1:2). C-3-G—cyanidin-3-glucoside; C-3-DG—cyanidin-3-dioxalylglucoside; Q—quercetin; EA—ellagic acid; ChA—chlorogenic acid; HC-1 and HC-2—two derivatives of hydroxycinnamic acid; P—phloretin; Ph—phlorizin hydrate. Values in the same row marked (a–g) with different superscripts are statistically different.

**Table 4:** Color parameters of formulated microparticles.

Samples	L*	a*	b*	ΔE	°h	C*
AF_B	48.13 ± 0.01 ^e^	19.06 ± 0.07 ^c^	11.23 ± 0.02 ^c,d^		29.81 ± 0.07 ^c^	22.59 ± 0.07 ^c,d^
AF_S_0.5	48.73 ± 0.06 ^d^	20.08 ± 0.06 ^a^	11.53 ± 0.02 ^c^	0.83	29.88 ± 0.10 ^c^	23.15 ± 0.04 ^b^
AF_S_1	50.86 ± 0.03 ^c^	20.11 ± 0.05 ^a^	11.80 ± 0.03 ^b^	2.84	30.40 ± 0.10 ^b^	23.32 ± 0.03 ^b^
AF_S_2	52.75 ± 0.04 ^b^	20.37 ± 0.02 ^a^	12.54 ± 0.04 ^a^	4.87	31.62 ± 0.11 ^a^	23.93 ± 0.01 ^a^
AF_T_0.5	48.69 ± 0.03 ^d^	19.46 ± 0.02 ^b^	11.12 ± 0.03 ^d^	0.59	29.73 ± 0.04 ^c^	22.43 ± 0.03 ^d^
AF_T_1	50.30 ± 0.07 ^c^	19.63 ± 0.02 ^b^	11.49 ± 0.01 ^c^	2.19	30.36 ± 0.02 ^b^	22.74 ± 0.01 ^c^
AF_T_2	55.14 ± 0.04 ^a^	17.98 ± 0.02 ^d^	10.25 ± 0.05 ^e^	7.26	29.69 ± 0.11 ^c^	20.70 ± 0.03 ^e^

AF_B—apple fiber/blackberry microparticles (control sample); AF—apple fiber; S—sucrose; T—trehalose; 0.5—ratio of apple fibers:disaccharide (1:0.5); 1—ratio of apple fibers:disaccharide (1:1); 2—ratio of apple fibers:disaccharide (1:2). Values in the same column marked (a–e) with different superscripts are statistically different.

**Table 5:** Volatile compounds (µg/kg) defined in blackberry juice (BJ), apple fiber (AF), and formulated microparticles ©, their odor threshold, and the flavor descriptor.

Volatiles	BJ	AF	C	RT	RI	OT	Descriptor
Hexanal	-	52.64 ± 1.56	+	5.13	800	20	green
Heptanal	0.72 ± 0.00	6.29 ± 0.10	+	10.76	897	3	green
D-limonene	7.37 ± 0.13	64.90 ± 1.15	+	19.41	1018	10	citrus
Citronellal	1.85 ± 0.02	24.40 ± 0.46	+	21.13	1051	25	floral
Linalool oxide	62.27 ± 1.54	-	-	22.19	1068	100	floral
Guaiacol	104.17 ± 1.80	-	+	23.17	1080	20	green
Linalool	23.39 ± 0.42	80.92 ± 4.45	+	23.96	1096	6	floral
Phenethyl alcohol	47.39 ± 0.48	-	+	24.6	1103	1000	floral
Menthol	13.86 ± 0.28	-	+	27.75	1167	920	minty
Nerol	20.25 ± 0.61	-	+	30.63	1222	290	citrus
Citral	17.02 ± 0.36	4.92 ± 0.21	+	32.64	1265	30	citrus
Perillyl alcohol	29.19 ± 1.98	3.71 ± 0.13	+	33.9284	1290	1660	green
Eugenol	31.19 ± 0.01	-	+	36.5930	1355	30	spicy
β-damascenone	11.90 ± 0.12	5.89 ± 0.24	+	37.6815	1381	10	floral
α-ionone	13.10 ± 0.44	1.64 ± 0.09	+	38.95	1420	0.6	floral
γ-ionone	4.18 ± 0.43	4.24 ± 0.19	+	40.18	1473	0.07	floral
β-ionone	2.58 ± 0.11	5.46 ± 0.43	+	40.34	1480	0.1	floral

“-”—not detected; “+”—detected; RT—retention time (min); RI—retention index; OT—odor threshold (µg/kg), http://www.leffingwell.com (accessed on 23 May 2022).

**Table 6:** Volatile compounds (µg/kg) in formulated microparticles.

Volatiles	AF_B	S_0.5	S_1	S_2	T_0.5	T_1	T_2
Hexanal	41.75 ± 0.23 ^f^	69.16 ± 3.28 ^e^	125.91 ± 0.19 ^d^	138.37 ± 0.65 ^b^	70.55 ± 0.71 ^e^	136.82 ± 0.44 ^c^	143.68 ± 0.47 ^a^
Heptanal	0.81 ± 0.00 ^e^	5.80 ± 0.01 ^d^	6.87 ± 0.06 ^c^	7.74 ± 0.09 ^b^	7.74 ± 0.04 ^b^	7.57 ± 0.12 ^b^	9.04 ± 0.63 ^a^
D-limonene	115.62 ± 2.11 ^b^	117.08 ± 0.45 ^b^	115.37 ± 0.75 ^b^	88.22 ± 0.47 ^e^	96.25 ± 1.24 ^d^	119.33 ± 1.33 ^b^	154.68 ± 0.95 ^a^
Citronellal	54.67 ± 0.66 ^b^	54.61 ± 0.66 ^b^	44.95 ± 0.10 ^c^	40.47 ± 1.07 ^d^	67.03 ± 0.47 ^a^	35.74 ± 0.60 ^e^	35.19 ± 0.55 ^e^
Guaiacol	14.99 ± 0.22 ^d^	16.51 ± 0.14 ^c^	14.45 ± 0.05 ^e^	12.53 ± 0.00 ^f^	21.73 ± 0.31 ^a^	18.14 ± 0.07 ^b^	16.94 ± 0.06 ^c^
Linalool	66.58 ± 0.68 ^b^	66.94 ± 1.57 ^b^	51.80 ± 0.03 ^d^	49.86 ± 0.06 ^e^	71.76 ± 0.48 ^a^	62.66 ± 0.21 ^c^	51.26 ± 0.90 ^d^
Phenethyl alcohol	22.19 ± 0.09 ^a^	19.98 ± 0.05 ^b^	11.73 ± 0.07 ^f^	9.71 ± 0.08 ^h^	17.99 ± 0.11 ^c^	13.92 ± 0.06 ^d^	12.94 ± 0.11 ^e^
Menthol	7.48 ± 0.09 ^a^	6.78 ± 0.01 ^c^	6.26 ± 0.02 ^d^	4.62 ± 0.15 ^f^	7.22 ± 0.03 ^b^	6.22 ± 0.03 ^d^	5.85 ± 0.05 ^e^
Nerol	10.21 ± 0.23 ^a^	10.01 ± 0.13 ^a^	6.38 ± 0.09 ^d^	3.40 ± 0.05 ^f^	8.79 ± 0.27 ^b^	6.75 ± 0.02 ^c^	6.12 ± 0.04 ^e^
Citral	13.53 ± 0.07 ^c^	13.97 ± 0.13 ^b^	9.76 ± 0.10 ^e^	9.43 ± 0.10 ^e^	16.92 ± 0.46 ^a^	13.00 ± 0.17 ^d^	13.49 ±0.06 ^c^
Perillyl alcohol	10.20 ± 0.25 ^b^	10.51 ± 0.00 ^b^	9.01 ± 0.05 ^c^	8.08 ± 0.11 ^d^	11.13 ± 0.15 ^a^	9.06 ± 0.03 ^c^	8.86 ± 0.16 ^c^
Eugenol	3.19 ± 0.16 ^c^	3.56 ± 0.13 ^b^	2.15 ± 0.02 ^e^	2.75 ± 0.01 ^d^	4.19 ± 0.30 ^a^	2.85 ± 0.03 ^d^	3.17 ± 0.19 ^c^
β-damascenone	8.29 ± 0.07 ^a^	6.68 ± 0.11 ^c^	5.69 ± 0.36 ^e^	4.70 ± 0.24 ^f^	7.58 ± 0.05 ^b^	6.02 ± 0.08 ^d^	5.62 ± 0.05 ^e^
α-ionone	2.61 ± 0.01 ^b^	2.81 ± 0.00 ^a^	2.65 ± 0.05 ^b^	1.73 ± 0.03 ^d^	2.83 ± 0.01 ^a^	2.63 ± 0.01 ^b^	2.27 ± 0.01 ^c^
γ-ionone	9.62 ± 0.14 ^b^	7.99 ± 0.19 ^c^	6.26 ± 0.08 ^d^	4.58 ± 0.21 ^e^	10.30 ± 0.06 ^a^	8.18 ± 0.02 ^c^	6.55 ± 0.04 ^d^
β-ionone	10.68 ± 0.13 ^a^	5.55 ± 0.04 ^d^	5.12 ± 0.04 ^e^	5.09 ± 0.10 ^e^	7.33 ± 0.12 ^b^	7.02 ± 0.03 ^c^	7.34 ± 0.04 ^b^

AF_B—apple fiber/blackberry microparticles (control sample); S—sucrose; T—trehalose; 0.5—ratio of apple fibers:disaccharide (1:0.5); 1—ratio of apple fibers:disaccharide (1:1); 2—ratio of apple fibers:disaccharide (1:2). Values in the same row marked (a–f) with different superscripts are statistically different.

**Table 7:** Odor activity values (OAVs) of blackberry juice (BJ), apple fiber (AF), and formulated microparticles.

Volatiles	BJ	AF	Samples						
			AF_B	S_0.5	S_1	S_2	T_0.5	T_1	T_2
Hexanal	0.00	2.63	2.09	3.46	6.30	6.92	3.53	6.84	7.18
Heptanal	0.24	2.10	0.27	1.93	2.29	2.58	2.58	2.52	3.01
D-limonene	0.74	6.49	11.56	11.71	11.54	8.82	9.63	11.93	15.47
Citronellal	0.07	0.98	2.19	2.18	1.80	1.62	2.68	1.43	1.41
Linalool oxide	0.62	0.00	0.00	0.00	0.00	0.00	0.00	0.00	0.00
Guaiacol	5.21	0.00	0.75	0.83	0.72	0.63	1.09	0.91	0.85
Linalool	3.90	13.49	11.10	11.16	8.63	8.31	11.96	10.44	8.54
Phenethyl alcohol	0.05	0.00	0.02	0.02	0.01	0.01	0.02	0.01	0.01
Menthol	0.02	0.00	0.01	0.01	0.01	0.01	0.01	0.01	0.01
Nerol	0.07	0.00	0.04	0.03	0.02	0.01	0.03	0.02	0.02
Citral	0.57	0.16	0.45	0.47	0.33	0.31	0.56	0.43	0.45
Perillyl alcohol	0.02	0.00	0.01	0.01	0.01	0.00	0.01	0.01	0.01
Eugenol	1.04	0.00	0.11	0.12	0.07	0.09	0.14	0.10	0.11
β-damascenone	1.19	0.59	0.83	0.67	0.57	0.47	0.76	0.60	0.56
α-ionone	21.83	2.73	4.35	4.68	4.42	2.88	4.72	4.38	3.78
γ-ionone	29.71	30.57	137.43	114.14	89.43	65.43	147.14	116.86	93.57
β-ionone	25.80	54.60	106.80	55.50	51.20	50.90	73.30	70.20	73.40

AF_B—apple fiber/blackberry microparticles; S—sucrose; T—trehalose; 0.5—ratio of apple fibers: disaccharide (1:0.5); 1—ratio of apple fibers:disaccharide (1:1); 2—ratio of apple fibers:disaccharide (1:2).

## Data Availability

The data sets generated and analyzed in the current study are available from the corresponding author upon reasonable request.
